# Endoscopic Cholecystoduodenostomy With Lumen-Apposing Metal Stent for Bile Duct Stricture in a Child with Neuroblastoma: A Case Report

**DOI:** 10.1055/a-2662-2339

**Published:** 2025-08-05

**Authors:** Maria Stella Cipriani, Liliana Piro, Federico Palo, Andrea Chiaro, Stefania Sorrentino, Paolo Gandullia, Andrea Parodi, Stefano Avanzini

**Affiliations:** 1 Pediatric Surgery Department, IRCCS, Istituto Giannina Gaslini, Genoa, Italy; 2 Dipartimento di Neuroscienze, Riabilitazione, Oftalmologia, Genetica e Scienze Materno-Infantili, University of Genoa, Genoa, Italy; 3 Pediatric Gastroenterology and Endoscopy Unit, IRCCS,Istituto Giannina Gaslini, Genoa, Italy; 4 Oncology Unit, IRCCS, Istituto Giannina Gaslini, Genoa, Italy; 5 Gastroenterology and Endoscopy Unit, Ospedale di Lavagna, Lavagna, Italy

**Keywords:** case report, neuroblastoma, common bile duct injury, cholecystoduodenostomy, endoscopy

## Abstract

We report the use of endoscopic cholecystoduodenostomy in a 6-year-old child to manage postanastomotic stricture of the common bile duct (CBD) secondary to an intraoperative injury sustained during the resection of an abdominal neuroblastoma (NB). The patient was diagnosed with stage M NB, characterized by dissemination to the bone marrow and vertebrae, and MYCN amplification. Following multiple cycles of chemotherapy and subsequent hematopoietic stem cell transplantation, the patient was scheduled for surgical resection. Preoperative imaging identified several image-defined risk factors, including infiltration of the porta hepatis and of the duodenopancreatic complex. During the dissection of the tumor, an incidental injury to the CBD occurred, which was subsequently repaired via end-to-end anastomosis. Seven months postoperatively, the patient presented with obstructive jaundice due to an anastomotic stricture, which was successfully managed through the placement of an endoscopic ultrasound-guided lumen-apposing metal stent (LAMS) between the dilated gallbladder and the duodenum. In our experience, endoscopic cholecystoduodenostomy constitutes a novel approach for addressing postoperative iatrogenic CBD strictures in pediatric patients. Further research is warranted to elucidate its benefits and risks as well as to evaluate its long-term efficacy and potential for broader application.

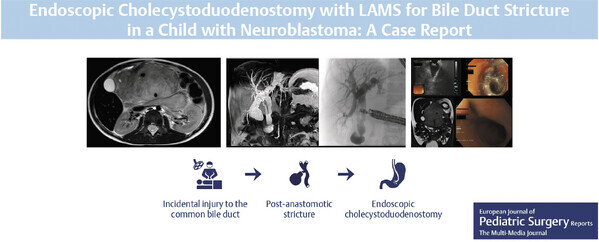

## Introduction

The management of postoperative adverse events in children affected by high-risk neuroblastoma (NB) is crucial for improving long-term quality of life and survival. Surgical resection of centrally located abdominal tumors often involves intricate dissection near vital structures, increasing the risk of bile duct injuries. Obstructive jaundice (OJ) secondary to biliary complications presents a critical challenge in children with high-risk NB. If left untreated, it can lead to liver failure, requiring timely interventions to ensure proper biliary drainage.

To our knowledge, this is the first case reporting the use of endoscopic cholecystoduodenostomy in a child as a solution for postanastomotic stricture of the common bile duct (CBD) secondary to intraoperative injury occurred during the resection of an abdominal NB.

## Case Report

A 6-year-old female patient was diagnosed with stage M abdominal NB with dissemination to the bone marrow and vertebrae, as well as MYCN amplification. The tumor was classified as high risk according to the International Neuroblastoma Risk Group (INRG) pretreatment classification.

The patient initially underwent seven cycles of chemotherapy due to a suboptimal response to induction therapy, followed by a double hematopoietic stem cell transplantation, in accordance with the International Society of Paediatric Oncology Europe Neuroblastoma Group HRNBL1 protocol.

**Fig. 1:**
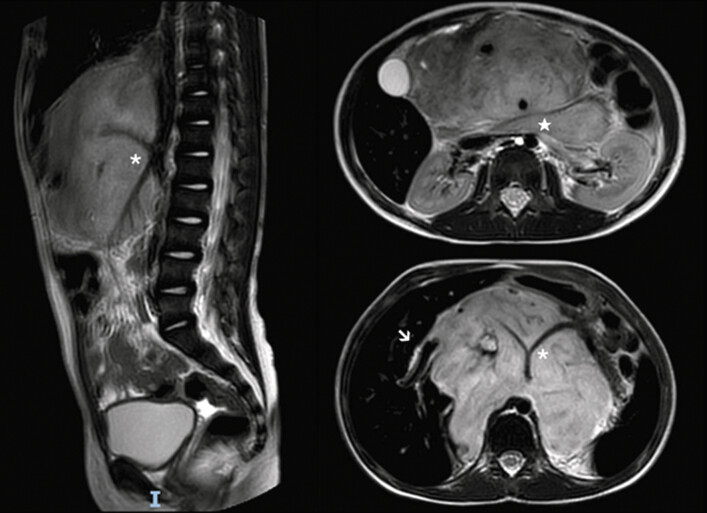
Preoperative Magnetic Resonance Imaging showing tumor infiltration of the porta hepatis (arrow), tumor encasement of the origins of the celiac axis and the superior mesenteric artery (asterisk), and tumor infiltration of the duodenopancreatic region (star).

After completing treatment, at the age of 7, she underwent laparotomic tumor excision at our institute. Preoperative magnetic resonance imaging (MRI) revealed several image-defined risk factors, including tumor infiltration of the porta hepatis, encasement of the celiac axis and superior mesenteric artery origins, and involvement of the duodenopancreatic region ( Fig. 1 ). During dissection, an incidental CBD injury occurred, which was repaired with end-to-end anastomosis and biliary drainage. Due to the tumor's complexity, only a partial resection was achieved, leaving significant macroscopic residual disease. Pathology revealed a predominance of Schwannian stroma (75%) with 25% neuroblastic cells, of which 85% were viable tumor and 15% showed postchemotherapy necrosis.

The patient was discharged on postoperative day 36 and subsequently underwent radiotherapy in accordance with the HRNBL protocol.

Six months after the surgery, MRI showed a stable residual tumor, persistent elevation of urinary catecholamines, and a negative bone marrow examination. Considering the tumor histology, the multidisciplinary tumor board decided against further treatment.

**Fig. 2:**
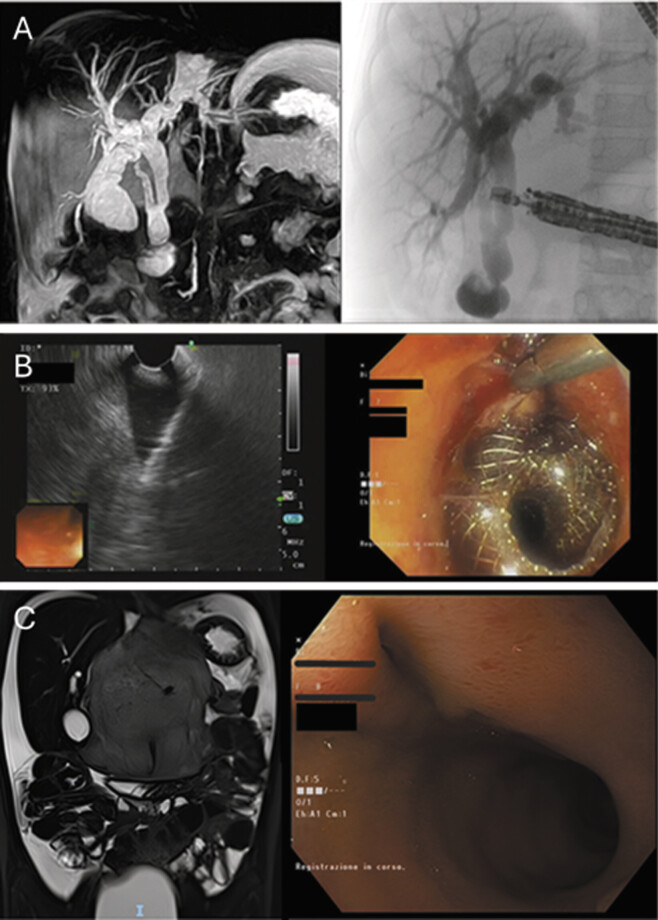
( **A** ) Ce-MRCP and ERCP performed 7 months after tumor excision, showing a CBD stricture. ( **B** ) EUS-guided cholecystoduodenostomy. ( **C** ) Postprocedure imaging demonstrating complete resolution of intrahepatic bile duct dilation, with endoscopy confirming a stable cholecystoduodenostomy. Ce-MRCP, contrast-enhanced magnetic resonance cholangiopancreatography; ERCP, endoscopic retrograde cholangiopancreatography; EUS, endoscopic ultrasound.

One month later, the patient presented with OJ and a progressive increase in total bilirubin levels, reaching 8.9 mg/dL. Contrast-enhanced magnetic resonance cholangiopancreatography demonstrated dilation of the left intrahepatic biliary system and gallbladder, along with complete obstruction of the distal CBD ( Fig. 2A ).

At 8 years old and weighing 20 kg, the patient underwent an endoscopic retrograde cholangiopancreatography (ERCP), which was unsuccessful due to tumor encasement of Vater's papilla. To restore biliary drainage, an endoscopic ultrasound (EUS)-guided cholecystoduodenostomy was performed using an Olympus GF-UCT160-AL5 ultrasound endoscope, with placement of a 10 × 10 Hot AXIOS lumen-apposing metal stent (LAMS) between the dilated gallbladder and duodenum ( Fig. 2B ). The procedure lasted approximately 3 hours, although the LAMS placement itself took only 20 minutes, most of the time was spent trying to overcome CBD stricture. The main challenge in LAMS positioning was identifying an adequate acoustic window, as the duodenum, already narrow due to the patient's small size, was significantly deformed by the tumor and the gallbladder insufficiently distended. To reduce the risk of incomplete flange deployment, the gallbladder was punctured with a fine-needle aspiration (FNA) needle and distended under EUS guidance using saline and contrast. This allowed precise LAMS placement under both EUS and fluoroscopic control. Due to the narrow duodenum, the distal flange could not be deployed under direct endoscopic vision; instead, it was released within the endoscope's channel and advanced into the duodenum using the introducer while retracting the endoscope. The endoscopic cholecystoduodenostomy resulted in bilirubin normalization and symptom resolution. Postoperative imaging confirmed normal contrast medium passage through the biliary tree ( Fig. 2C ). According to the Clavien–Dindo classification, this complication is classified as grade 3b.

At the 3-month follow-up, the patient was in good general condition with normal bilirubin levels. Endoscopic and radiological assessments revealed a stable tumor residue and the spontaneous dislodgment of the LAMS device, alongside a stabilized cholecystoduodenostomy ( Fig. 2 ).

## Discussion Which Closes with a Conclusion

This case report demonstrates the potential of advanced endoscopic interventions to address challenging biliary complications in pediatric oncology. The use of LAMS for endoscopic cholecystoduodenostomy, although established in adult populations, is underreported in children, particularly in cases of CBD strictures resulting from complex oncological surgeries.

According to the INRG staging system, the extent of involvement of vital structures influences staging, therapeutic decisions, and prognosis in NB. Tumor encasement of the biliary tract is a rare event and is frequently associated with incomplete resection of the mass.

Management of biliary tract stricture traditionally involves stent placement or surgical biliodigestive bypass. However, these methods may be constrained in cases where crossing the papilla of Vater is not feasible or when the tumor encases the CBD. Furthermore, performing a biliodigestive bypass in a patient who has previously undergone surgery in the duodenopancreatic region and has a residual tumor—when feasible—carries a high risk of intra- and postoperative complications, including visceral injury, major bleeding, and anastomotic dehiscence.

The first use of endoscopic transduodenal drainage of the gallbladder using a pigtail biliary stent was described in 2006. 1 The LAMS was first described in animal studies in 2011, 2 and its feasibility for EUS-guided gallbladder drainage (EUS-GBD) in humans was demonstrated in 2014. 3

Currently, EUS-GBD using an LAMS is a frequently adopted technique in adults with acute cholecystitis or biliary obstruction due to malignancy or cholelithiasis outside of the cystic duct, particularly when invasive procedures are not feasible due to the patient's poor clinical status. This technique has been included as a minimally invasive treatment option in the updated Tokyo Guidelines for acute cholecystitis. 4 Moreover, it has been reported that EUS-GBD is an effective and secure rescue therapy for distal malignant biliary obstruction following failure of ERCP and/or EUS-guided bile duct drainage. 5

Literature on the management of OJ in NB includes temporary cholecystostomy tube placement, percutaneous transhepatic biliary drainage, and endoscopic internal biliary drainage. 6 7 8 However, the application of cholecystoduodenostomy in pediatric oncology remains underexplored. To our knowledge, this case represents the first documented use of endoscopic cholecystoduodenostomy in managing OJ in a child with NB.

The advantages of LAMS include being less invasive than surgery and causing less psychological impact than an external biliary drain. Specifically, in our patient with a poor-prognosis tumor who had already undergone major surgery, biliary drainage with LAMS allowed minimally invasive treatment of jaundice despite the failure of ERCP, while avoiding the placement of an external biliary drain. Although the risk of dislodgement exists, selecting a stent diameter compatible with the patient's anatomy—10 mm in this case—helps minimize this risk. Both dislodgement and obstruction are manageable thanks to the possibility of repeating the LAMS placement or replacing the stent.

In our experience, the innovative approach of endoscopic cholecystoduodenostomy has proven effective in bypassing CBD stricture and alleviating associated jaundice. This minimally invasive technique facilitated rapid clinical improvement and significant reduction in bilirubin levels, offering a promising alternative to more invasive traditional surgical techniques.

In conclusion, advanced endoscopic approaches, such as cholecystoduodenostomy with LAMS, represent a notable advancement in managing iatrogenic CBD strictures, in pediatric patients. This case underscores the need for further studies to evaluate the efficacy, safety, and long-term outcomes of this innovative approach in children.

## Notes

Importance for a Pediatric Surgeon This article describes a novel application of endoscopic cholecystoduodenostomy to treat obstructive jaundice caused by a postanastomotic stricture of the common bile duct, following neuroblastoma surgery in a child.
